# Assessing antidepressant exposure and response using UK electronic health record data

**DOI:** 10.23889/ijpds.v9i5.2488

**Published:** 2024-09-18

**Authors:** Matthew Iveson, Emily Ball, Arlene Casey, Matus Falis, Andrew McIntosh, Heather Whalley

**Affiliations:** 1 The University of Edinburgh

## Objective and Approach

Many people do not respond to antidepressants and our poor mechanistic understanding of antidepressant response has hampered efforts to personalise treatment. Understanding how and when antidepressants work at an individual level requires real-world antidepressant exposure and response data, at scale.

To date, studies have rarely had access to sufficiently detailed clinical data at the scales needed to derive such phenotypes and track them longitudinally. Indeed, many studies use research-based measures and phenotypes that are not commonly used in clinical settings, making translation to clinically-applicable tools difficult.

This study aims to develop novel measures of antidepressant treatment exposure and response by capitalising on the variety and volume of UK clinical records. We identify cases of depression from national primary and secondary care records, and derive longitudinal antidepressant treatment episodes from national community dispensing data.

## Results

Focusing on the Generation Scotland cohort (N = 21,000), we describe common antidepressant treatment trajectories and identify changes in medication, dose and strength. We examine the frequency of drug switching, augmentation, and drug stability, contrasting these to other UK linked cohorts. We also describe the demographic characteristics of those defined as treatment resistant and those identified as in drug maintenance.

## Conclusions

Linked electronic records provide the opportunity to track antidepressant exposure and response longitudinally. Changes in treatment can provide new measures that help to better understand treatment resistance and maintenance.

## Implications

By better tracking antidepressant response, the present study helps inform mechanistic research to understand treatment effectiveness and ultimately to develop personalised treatments.

